# Machine Learning to Identify Critical Biomarker Profiles in New SARS-CoV-2 Variants

**DOI:** 10.3390/microorganisms12040798

**Published:** 2024-04-15

**Authors:** Christoph Schatz, Ludwig Knabl, Hye Kyung Lee, Rita Seeboeck, Dorothee von Laer, Eliott Lafon, Wegene Borena, Harald Mangge, Florian Prüller, Adelina Qerimi, Doris Wilflingseder, Wilfried Posch, Johannes Haybaeck

**Affiliations:** 1 Tyrolpath Obrist Brunhuber GmbH, 6311 Zams, Austrialudwig.knabl@tyrolpath.at (L.K.); 2 Institute of Pathology, Neuropathology and Molecular Pathology, Medical University of Innsbruck, Muellerstrasse 44, 6020 Innsbruck, Austria; adelina.kjerimi@i-med.ac.at; 3 Laboratory of Genetics and Physiology, National Institute of Diabetes and Digestive and Kidney Diseases, National Institutes of Health, Bethesda, MD 20892, USA; hyekyung.lee@nih.gov; 4 Department Life Sciences, IMC University of Applied Sciences Krems, 3500 Krems, Austria; rita.seeboeck@stpoelten.lknoe.at; 5 Clinical Institute of Pathology, University Hospital St. Poelten, Karl Landsteiner University of Health Science, 3100 St. Poelten, Austria; 6 Institute of Virology, Medical University of Innsbruck, Peter-Mayr-Strasse 4b, 6020 Innsbruck, Austriawegene.borena@i-med.ac.at (W.B.); 7 Institute of Hygiene and Medical Microbiology, Medical University of Innsbruck, Schöpfstrasse 41, 6020 Innsbruck, Austriadoris.wilflingseder@i-med.ac.at (D.W.); wilfried.posch@i-med.ac.at (W.P.); 8 Clinical Institute for Medical and Chemical Laboratory Diagnosis (CIMCL), Medical University of Graz, Auenbruggerplatz 15, 8036 Graz, Austria; 9 Department of Pathobiology, Infectiology, Veterinary University of Vienna, Veterinärplatz 1, 1210 Vienna, Austria; 10 Department of Pathology, Saint Vincent Hospital Zams, 6511 Zams, Austria; 11 Diagnostic and Research Center for Molecular BioMedicine, Institute of Pathology, Medical University of Graz, 8010 Graz, Austria; 12 Department of Pathology, Laborteam, 9403 Goldach, Switzerland; 13 Department of Pathology, University Medical Centre Maribor, 2000 Maribor, Slovenia

**Keywords:** SARS-CoV-2, vaccination state, variants, Alpha, Alpha + E484K, Beta, Omicron, z-scores, PC algorithm, precision, recall, F1 score, machine learning, Restricted Boltzmann Machine neural network

## Abstract

The global dissemination of SARS-CoV-2 resulted in the emergence of several variants, including Alpha, Alpha + E484K, Beta, and Omicron. Our research integrated the study of eukaryotic translation factors and fundamental components in general protein synthesis with the analysis of SARS-CoV-2 variants and vaccination status. Utilizing statistical methods, we successfully differentiated between variants in infected individuals and, to a lesser extent, between vaccinated and non-vaccinated infected individuals, relying on the expression profiles of translation factors. Additionally, our investigation identified common causal relationships among the translation factors, shedding light on the interplay between SARS-CoV-2 variants and the host’s translation machinery.

## 1. Introduction

Coronaviruses belong to the order of Nidovirales and infect mammals and birds [1,2]. The positive single-stranded RNA virus SARS-CoV-2 is related to MERS-CoV and SARS-CoV and causes acute and severe respiratory symptoms. In contrast, other widespread coronaviruses from the genus *Alphacoronavirus* cause seasonally clustered, usually mild, infections of the respiratory and gastrointestinal tracts in humans [3,4]. SARS-CoV-2 enters the cell via ACE2 or alternative receptors like C-type lectins, CD147, NRP1, or others [5]. After membrane fusion, the viral RNA is released in the cytoplasm, where non-structural proteins form replication organelles. Viral structural proteins and genomic RNA synthesized at the replication sites are translocated to the ER–Golgi intermediate compartment (ERGIC), where virus assembly and budding occur [6]. Among all the host cell structures and functionalities, viruses depend on the protein synthesis machinery inter alia. Viruses can directly target ribosomal proteins, ribosomal biogenesis factors, and translation initiation factors, emphasizing the synthesis of viral proteins and repressing the translation of host mRNAs [7,8,9,10,11]. One hotspot of the betacoronavirus SARS-CoV-2 after the outbreak in Wuhan [12,13,14] was Ischgl in early March 2020 [15].

The translation of RNA into proteins is a major level of regulating gene expression and is important for homeostasis and rapid intracellular responses to environmental triggers [6]. EIFs are required to assemble the 80S ribosome consisting of mRNA and initiator tRNA as well as 40S and 60S ribosomal subunits [16]. The canonical translation mechanism used by the majority of human RNAs depends on interaction with the 5′-cap structure and is therefore also referred to as cap-dependent translation. Here, the eIF4F complex, comprising the cap-binding eIF4E, DNA helicase eIF4A, and scaffolding eIF4G, binds mRNA for translation initiation. eIF3 interacts with eIF4G and the ribosome, bringing them together in a complex that further contains eIF2 and an initiator met-tRNA. The process is enhanced by the interaction of eIF4G with the poly-A binding protein PABP, a process that circularizes the mRNA [17].

However, phosphorylated eIF2α results in protein shutdown and cell death [18]. Moreover, the P38MAPK and ERK pathways are stimulated by SARS-CoV-2, enabling EIF4E to enhance translation following phosphorylation by p38 MAPK and ERK1/2-mediated phosphorylation of Mnk1 [19]. SARS-CoV-2 interactions were reported to be associated with *EIF4H* [20], *EIF4G* [21], and *EIF4E* [21]. Additionally, an association of eIF3 and SARS-CoV-2 was found [22].

Since eIFs are targeted by many viruses upon cell entry, providing a favorable condition for their own replication [23], we thereby investigated the expression differences in the eukaryotic translation factors and mTOR between different variants. We found evidence that eukaryotic translation factors are regulated to different levels between the virus variants (Alpha, Alpha + E484K, Beta, and Omicron) and also upon vaccination, representative of the grade of severity. Interrupting the translation machinery might have a beneficial impact on the disease course.

## 2. Materials and Methods

### 2.1. Data and Samples

The data for the analyses were based on mRNA expression upon the response of the cells by SARS-CoV-2 infections.

Total RNA was extracted from the buffy coat (white blood cells) of whole blood samples. A purification was executed using a Maxwell RSC simply RNA Blood Kit. The quality and concentration of the RNA were evaluated with an Agilent Bioanalyzer 2100 device (Agilent, Santa Clara, CA, USA).

Maxwell RSC simply RNA Blood purification kits were used for extracting RNA from the patients’ blood. Reverse transcriptase with random priming was used to create cDNA. Primers with sequences from ARTICnetwork were employed to generate 400 bp amplicons in 2 varying PCR pools. Following the amplification and merging of the pools, libraries were established with a QIASeq FX DNA Library UDI kit (Qiagen, Hilden, Germany). Illumina NextSeq 500/550 was used for sequencing, combined with 149 bp paired-end reads and 10 bp indices (Illumina, San Diego, CA, USA). An assembly of viral sequences was performed using CLC Genomics workbench v20.0.3 (Qiagen, Hilden, Germany). As a reference genome, SARS-CoV-2 Wuhan-Hu-1 was used (Accession NC_045512.2). FASTA files from http://cov-lineages.org/ (accessed on 20 November 2023) assisted the identification of SARS-CoV-2 variants. Poly-T oligo hybridization assisted the Poly-A-containing mRNA purification from 1 µg of RNA. SuperScript III (Invitrogen, Waltham, MA, USA) was used to synthesize cDNA. TruSeq Stranded mRNA Library Prep Kits (Illumina, San Diego, CA, USA, RS-20020595) were used to prepare the libraries for sequencing. Paired-end sequencing was performed with NovaSeq 6000 (Illumina, San Diego, CA, USA) with a yield of 190 million reads per sample.

The gene names are written italic, and the protein names are written standard.

### 2.2. Patient Cohort

Samples from patients were pooled and compared with healthy individuals (not asymptomatic, never infected, recovered) to obtain log2fold changes (Table 1).

### 2.3. Statistical Analyses

Paredes et al. stated that the Beta variant led, percentual, to more hospitalizations than the Alpha and Omicron variants, with the Omicron variant being the least severe [24]. Under the assumption of severity, Alpha, Alpha + E484K, and Omicron were compared to Beta, and samples with vaccination were compared to samples without vaccination (Table 2).

The z-scores were calculated using the following formula:

(1) $$z-score\;:=\frac{x-µ}{\sigma },$$

where µ is considered the mean of the group, σ is the standard deviation, and *x* is the datapoint, i.e., the median of the other group to be compared.

For calculating the precision and recall, the genes of the samples of each variant were compared with the same genes of the Beta variant, and the genes of each sample without vaccination were compared with the same genes of the vaccination samples. Log2fold changes were used.

*True Positive (TP): current value Beta variant/Unvaccinated > arithmetic mean other variant/Vaccinated* *False Positive (FP): current value Beta variant/Unvaccinated < arithmetic mean other variant/Vaccinated* *True Negative (TN): arithmetic mean Beta variant/Unvaccinated > current value other variant/Vaccinated* *False Negative (FN): arithmetic mean Beta variant/Unvaccinated < current value other variant/Vaccinated* (2)

Precision was calculated according to the following formula:

(3) $$precision\;:=\frac{TP}{TP+FP},$$

Recall was calculated using the following formula:

(4) $$recall\;:=\frac{TP}{TP+FN},$$

The *F*1 score was calculated as follows:

(5) $$F1\;score:=\frac{2\cdot precision\cdot recall}{precision+recall},$$

The following genes were extracted for statistical analysis: *EEF1DP3*, *EEF1E1*, *EIF1*, *EIF2S3*, *EIF3I*, *EIF4A2*, *EIF4G1*, *EIF4G2*, *EIF4H*, *EIF5*, *EIF5A*, *EIF4E*, *EIF1AX*, *EIF3K*, *EIF3M*, *EIF1AY*, *EEF1B2*, *MRRF*, *EIF6*, *EIF3A*, *EIF3E*, *EIF3L*, *EEF1A1*, *EIF4B*, *EEF1D*, *EIF2B5*, *EIF2B4*, *EIF2S2*, *EIF3D*, *EIF3H*, *EIF5A2*, *EIF2A*, *EEF2*, *EEF1G*, *EIF3C*, *EIF3G*, *EIF2B1*, *EIF2S1*, *EIF5B*, *EIF2B3*, *EIF3J*, *MTOR*, *EIF3B*, *EEF1A2*, and *EIF2B2*.

The package pcalg [25] (version 2.7.9) was used to create to a directed acyclic graph (DAG) using the functions skeleton and pc with gaussCItest to test for conditional independence and an alpha of 0.01 based on the log2fold changes.

The machine learning approach used in this study was based on the ML DotNet framework [26] (version 4.0.30319). The matrix of log2fold changes for each of the genes was used as the feature (type single) and the variant (type string, categorical), respectively, and the vaccination state (type string, categorical) was used as label for prediction. Finding the best model run took 10 min. The highest accuracy was achieved with the trainer LbfgsLogisticRegressionOva (prediction of SARS-CoV-2 variant). FastTreeOva led to the highest accuracy for the prediction of the vaccination state.

For the Restricted Boltzmann Machine (RBM) neural network, the R package [27] with the function darch (version 0.13.0) was used, including the following parameters: epochs = 50, layers (10, 10, 1), stopClassErr = 0, and retainData = TRUE. The CrossTable function was called using the R package gmodels (version 2.18.1.1).

### 2.4. Groups

The following samples were pooled to variant groups and to vaccination state groups of infected individuals. Samples were collected, and the data were obtained in the context of the following studies [28,29,30].

## 3. Results

**Figure 1:**
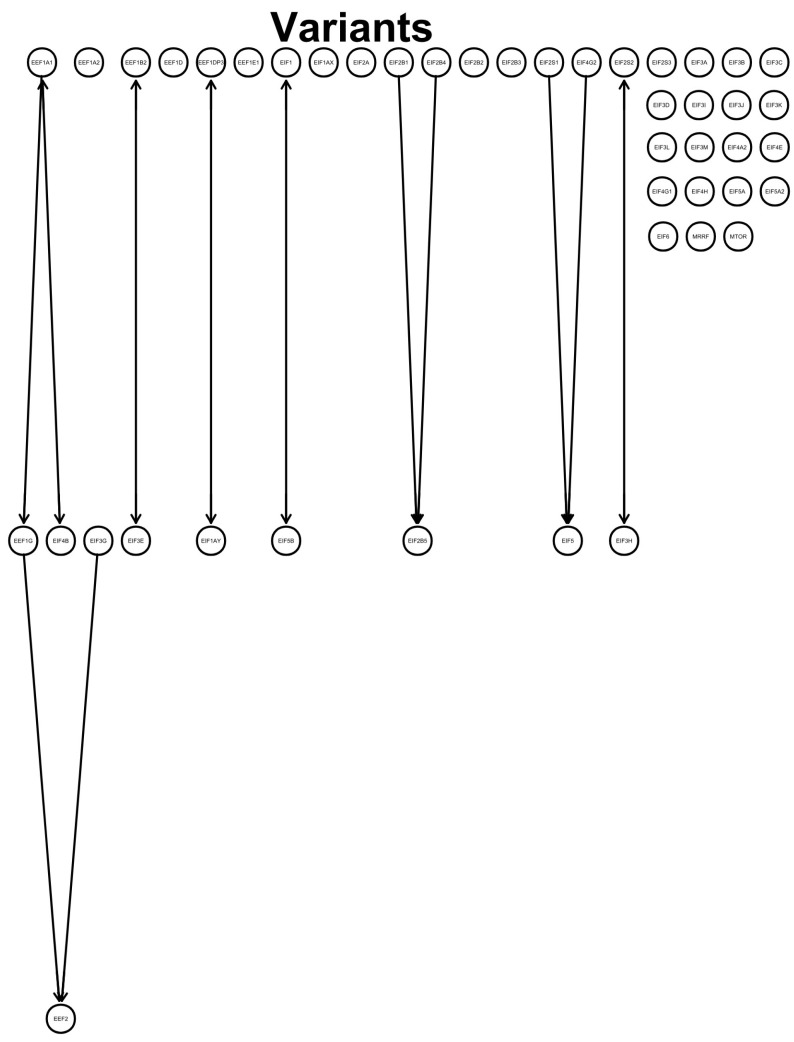
Graph: directed acyclic graph (DAG) shows a defined directed flow (lines and arrows) between the investigated genes. Variables were tested for conditional independence based on log2fold changes using pooled SARS-CoV-2 variants that led to directions between variables. Each gene expression (circle with gene symbol) was tested against the others for conditional independence. Found relations indicate a flow from one gene to another, as indicated by an arrowhead towards the target variable. No connected line represents no found connection between the variables, and arrowheads both from the next variable to the previous variable and from the previous variable to the next variable indicate an unmeasured confound.

Testing for conditional independence between the genetic expressions of eukaryotic translation factors revealed a direction from the elongation factors *EEF1A1* to *EEF1G* and from *EEF1G* to *EEF2*. *EIF3G* pointed to *EEF2*. *EIF2B1* and *EIF2B4* directed to *EIF2B5*, and *EIF2S1* and *EIF4G2* pointed to *EIF5*. Unmeasured confounds were found for *EEF1A1* and *EIF4B*, *EEF1B2* and *EIF3E*, *EEF1DP3* and *EIF1AY*, *EIF1* and *EIF5B*, and *EIF2S2* and *EIF3H*. No directions between variables were obtained for *EEF1A2*, *EEF1D*, *EEF1E1*, *EIF1AX*, *EIF2A*, *EIF2B2*, *EIF2B3*, *EIF2S3*, *EIF3A*, *EIF3B*, *EIF3C*, *EIF3D*, *EIF3I*, *EIF3J*, *EIF3K*, *EIF3L*, *EIF3M*, *EIF4A2*, *EIF4E*, *EIF4G1*, *EIF4H*, *EIF5A*, *EIF5A2*, *EIF6*, *MRRF*, and *MTOR* (Figure 1).

### Vaccinated Samples Resulted in z-Scores Higher Than 1 for EIF1AY

**Figure 2:**
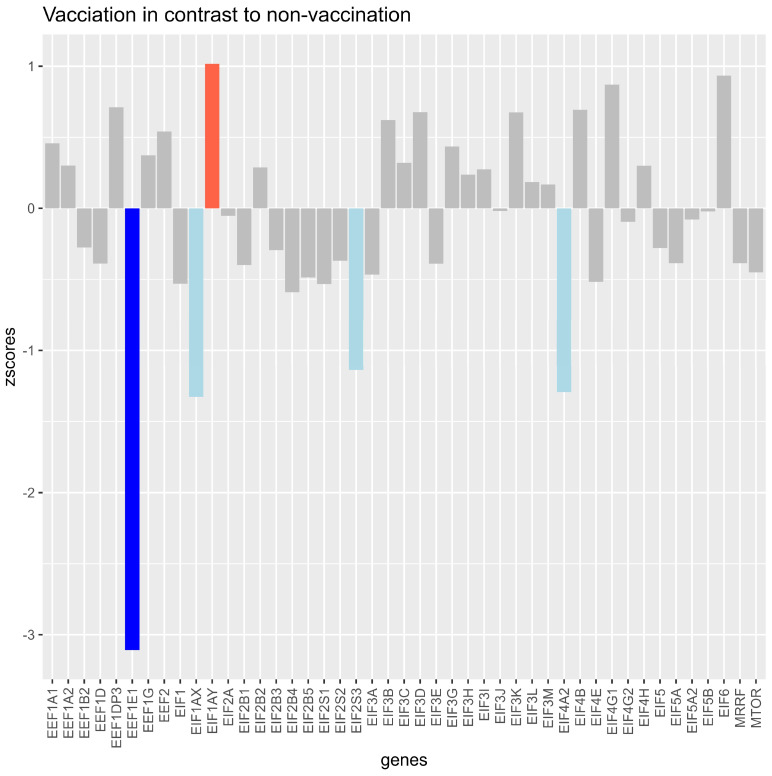
Z-scores of log2fold changes in translation factors and mTOR between samples of unvaccinated versus vaccinated samples (median datapoint), with all variants pooled together. The bars show how much the expression of the vaccinated group differed from the expression of the unvaccinated group for each investigated gene. Light blue bars indicate that the z-score was between −1 and lower than −2 for the gene, indicating a standardized lower expression of the vaccinated group for the gene. The dark blue bar indicates a z-score higher than −1, showing that the expression of the vaccinated group was lower than −2 compared with the unvaccinated group for the gene. The light red bar indicates that the expression (z-score higher than 1 and lower than 2) for the gene was higher in the vaccinated group.

*EIF1AX*, *EIF2S3*, and *EIF4A2* showed a decrease of more than −1, and *EEF1E1* led to a z-score of lower than −2 compared with the unvaccinated samples (Figure 2).

The highest precisions and recalls were calculated for *EEF1E1*, *EIF1*, *EIF2S3*, *EIF3I*, *EIF4A2*, *EIF4G2*, *EIF5*, and *EIF5A* (Beta compared with Alpha), reflecting generally higher expressions of the mRNAs of Beta, thus reflecting generally higher expressions of these mRNAs upon a SARS-CoV-2 Beta infection. *EEF1E1*, *EIF1*, *EIF2S3*, and *EIF3I* revealed the highest precisions when the Beta variant was compared with the Alpha variant in combination with the mutation E484K. Beta compared with Omicron revealed the highest precisions and recalls for *EEF1DP3*, *EIF1*, *EIF3I*, *EIF4G1*, *EIF4G2*, *EIF4H*, *EIF5*, *EIF4E*, and *EIF5A*.

In contrast, the lowest values of precisions and recalls were retrieved for *EIF2B4*, *EIF3B*, and *EEF1A2* (Beta compared with Alpha). *EIF6*, *EEF1A2*, *EIF2S1*, *EIF3A*, *EIF3B*, *EIF3G*, and *EIFEG* showed the lowest precisions and recalls after Beta was compared with Alpha, including the mutation *E484K*. Beta versus Omicron revealed the lowest precision and recall for *EEF1B2*, *EIF3C*, *EIF3E*, *EEF1G*, *EEF2*, *EIF3B*, *EIF3G*, and *EIF5A2*.

The lowest precisions and recalls were obtained for *EIF2A*, *EIF3D*, *EIF4G1*, and *EIF4H* when the unvaccinated samples were compared with the vaccinated samples.

**Figure 3:**
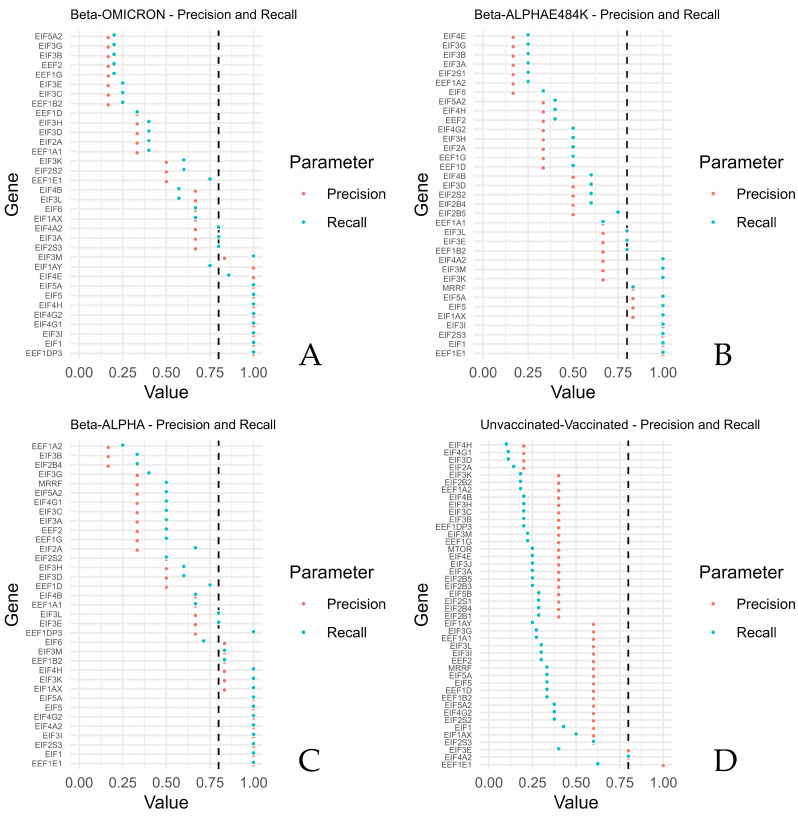
Precision against recall for the translation factors and mTOR, grouped by comparisons of Beta versus ALPHA, Beta versus ALPHA and E484K, Beta versus OMICRON, and unvaccinated versus vaccinated. For each sample of the compared groups, higher values indicate that the expression of the unvaccinated group was more often higher than the vaccinated group, respectively, and that the expression of the Beta group was more often higher than the expression of each other group (ALPHA, E48K, or OMICRON) for the investigated genes. The figure shows the precision and recall per gene for (**A**) Beta–OMICRON, (**B**) Beta–ALPHAE484K, (**C**) Beta–ALPHA and (**D**) unvaccinated–vaccinated.

High precisions and recalls were calculated for *EIF4A2* and *EEF1E1* when samples of the unvaccinated group were compared with samples of the vaccinated group (Figure 3). Supplementary Table S1 shows the F1 scores.

With machine learning, an accuracy of 0.75 based on the log2fold changes in the different translation factors and *MTOR* to predict the variant type was archived. Using the log2fold changes for the prediction of the vaccination status resulted in an accuracy of 0.60.

By using a Restricted Boltzmann Machine neural network, Beta (6/6) and Omicron (4/4) could be classified correctly. A distinction between Alpha and Alpha + E484K resulted in a false-classifying of Alpha as Alpha + E484K and vice versa.

## 4. Discussion

Despite extensive research efforts, an effective therapeutic intervention targeting the causative agent of COVID-19 remains elusive. Furthermore, there is an ongoing necessity for research to elucidate the underlying factors that drive the progression towards severe disease in COVID-19. The disruption of certain translation factors at the fundamental level of essential protein synthesis may prove adequate in impeding viral replication and propagation.

Viewed from the perspective of the Beta variant, the expression of the translation factors *EIF1* and *EIF3I* was higher compared with the Alpha, Alpha + E484K, and Omicron variants. Additionally, the Beta variant harbored a higher expression of *EEF1E1* and *EIF2S3* compared with both Alpha variants. The Beta variant compared with the Omicron variant revealed a higher expression of *EEF1DP3*, *EIF4G1*, *EIF4G2*, *EIF4H*, *EIF5*, and *EIF5A*, whereas *EIF5* and *EIF5A* were also expressed lower in the Alpha variant compared to the Beta variant (Figure 3).

Upon infection, the expression of *EIF2A*, *EIF3D*, *EIF4G1*, and *EIF4A* was increased (Figure 3).

Differences in eukaryotic translation factors between vaccinated and unvaccinated individuals were shown with z-scores higher and lower than 1 (Figure 2). Utilizing conditional independence, some directions and associations of the translation factors were found upon infection with the SARS-CoV-2 variants (Figure 1). Translation factors were differently expressed by counting true positives, true negatives, false positives, and false negatives and by deriving the precision and recall from the information (Figure 3).

The expression differences in some of the eukaryotic translation factors could be associated with a diverging severity response, respectively, with subtle differences in utilizing the extent of translation factors involved during the infection, and they could lead to a characteristic signature for identifying the variants and the clinical outcome.

The depicted directed effect of *EIF5* (Figure 1) could be reflected by the high precision and recall of the Beta variant versus the Alpha and Omicron variants.

The machine learning (ML DotNet) approach performed relatively well using all the translation factors and *MTOR* as variables to predict the variant. In three out of four cases, the classification was correct, and the reliability of a correct discrimination was supported by the Restricted Boltzmann Machine neural network, which only failed to differentiate between the Alpha and Alpha E484K variants, which have genetically more in common than the Beta and Omicron variants. The rate of accurately classifying the state of the translation factors and *MTOR* was only 60%. Vaccinations could have a dampening effect on the extent of the translation machinery, and the differences between the percentage of hospitalizations depending on the variants could be reflected by the translation factor profile. We suggest that a more severe course upon infection could lead to a stronger response with a sharper increase in some of the translation factors.

The limitations of this study include the low sample size consisting of relatively many groups with fewer samples (confounding), leading to a requirement of pooling. Correlations and inference tests were not applicable based on the low sample size. *EIF1AX* and *EIF2S3* are on the X-chromosome, and *EI1AY* is on the Y-chromosome.

Further research and more samples are required for clearer distinctions.

## Figures and Tables

**Table 1:** Pooled groups, group size, number (n) of male and female patients, and the median age of the groups and, if available, the severity and the days after a positive PCR result. The healthy group consisted of 30 individuals (median: 72 years, male: 4, female: 26).

Pooled Group	n Patients	n Male, n Female	Median Age	Severity
Alpha 1	31	15, 16	67	-
Alpha 2	29	13, 16	72	-
Alpha 3	5	4, 1	65	-
Alpha + EK 1	13	8, 5	74	-
Alpha + EK 2	10	7, 3	77	-
Alpha + EK 3	7	4, 3	80	-
Beta unvaccinated 1	5	1, 4	62	0 mild 3 moderate 2 severe
Beta unvaccinated 2	5	1, 4	62	-
Beta unvaccinated 3	4	1, 3	68	-
Beta vaccinated 1	4	2, 2	82	1 mild 0 moderate 3 severe
Beta vaccinated 2	3	2, 1	80	-
Beta vaccinated 3	3	2, 1	80	-
Omicron vaccination 1	22	-	-	-
Omicron vaccination 2	21	-	-	-
Omicron unvaccinated 1	44	-	-	-
Omicron unvaccinated 2	41	-	-	-

**Table 2:** Gene expression groups for statistics with log2foldchanges. Sixteen groups, with each group consisting of multiple samples, were used.

Sample Number	Variant	Vaccination State
1	Alpha	Unvaccinated
2	Alpha	Unvaccinated
3	Alpha	Unvaccinated
4	Alpha + E484K	Unvaccinated
5	Alpha + E484K	Unvaccinated
6	Alpha + E484K	Unvaccinated
7	Beta	Unvaccinated
8	Beta	Unvaccinated
9	Beta	Unvaccinated
10	Beta	Vaccinated
11	Beta	Vaccinated
12	Beta	Vaccinated
13	Omicron	Unvaccinated
14	Omicron	Unvaccinated
15	Omicron	Vaccinated
16	Omicron	Vaccinated

## Data Availability

Data are contained within the article and Supplementary Materials.

## Supplementary Materials

The following supporting information can be downloaded at: https://www.mdpi.com/article/10.3390/microorganisms12040798/s1, Supplementary Table S1: F1 scores of the Beta variant versus the Alpha variant. Alpha + E484K and Omicron variant and unvaccinated versus vaccinated.
