# Supplementary material for: Machine Learning to Identify Critical Biomarker Profiles in New SARS-CoV-2 Variants
Source: Microorganisms. 2024 Apr 15;12(4):798. doi: 10.3390/microorganisms12040798 (PMC11052335; doi:10.3390/microorganisms12040798)
Supplement: Supplementary file 1 [file microorganisms-12-00798-s001.zip › microorganisms-2880356-supplementary.pdf]

Supplementary Table S1. F1 scores of the Beta versus the Alpha variant, Alpha + E484K versus Omicron variant, and unvaccinated versus vaccinated

Gene	Comparison	F1 score
EEF1DP3	Beta-OMICRON	1
EEF1E1	Beta-ALPHAE484K	1
EEF1E1	Beta-ALPHA	1
EIF1	Beta-ALPHAE484K	1
EIF1	Beta-ALPHA	1
EIF1	Beta-OMICRON	1
EIF2S3	Beta-ALPHAE484K	1
EIF2S3	Beta-ALPHA	1
EIF3I	Beta-ALPHAE484K	1
EIF3I	Beta-ALPHA	1
EIF3I	Beta-OMICRON	1
EIF4A2	Beta-ALPHA	1
EIF4G1	Beta-OMICRON	1
EIF4G2	Beta-ALPHA	1
EIF4G2	Beta-OMICRON	1
EIF4H	Beta-OMICRON	1
EIF5	Beta-ALPHA	1
EIF5	Beta-OMICRON	1
EIF5A	Beta-ALPHA	1
EIF5A	Beta-OMICRON	1
EIF4E	Beta-OMICRON	0.923076923
EIF1AY	Beta-OMICRON	0.857142857
EEF1E1	Unvaccinated-Vaccinated	0.769230769
EIF1AX	Beta-ALPHAE484K	0.909090909
EIF1AX	Beta-ALPHA	0.909090909
EIF3K	Beta-ALPHA	0.909090909
EIF3M	Beta-OMICRON	0.909090909
EIF4H	Beta-ALPHA	0.909090909
EIF5	Beta-ALPHAE484K	0.909090909
EIF5A	Beta-ALPHAE484K	0.909090909
EEF1B2	Beta-ALPHA	0.833333333
EIF3M	Beta-ALPHA	0.833333333
MRRF	Beta-ALPHAE484K	0.833333333
EIF6	Beta-ALPHA	0.769230769
EIF4A2	Unvaccinated-Vaccinated	0.8
EIF3E	Unvaccinated-Vaccinated	0.533333333
EEF1DP3	Beta-ALPHA	0.8
EIF3K	Beta-ALPHAE484K	0.8
EIF3M	Beta-ALPHAE484K	0.8
EIF4A2	Beta-ALPHAE484K	0.8
EEF1B2	Beta-ALPHAE484K	0.727272727
EIF2S3	Beta-OMICRON	0.727272727
EIF3A	Beta-OMICRON	0.727272727
EIF3E	Beta-ALPHAE484K	0.727272727
EIF3E	Beta-ALPHA	0.727272727
EIF3L	Beta-ALPHAE484K	0.727272727
EIF3L	Beta-ALPHA	0.727272727
EIF4A2	Beta-OMICRON	0.727272727

EEF1A1	Beta-ALPHAE484K	0.666666667
EEF1A1	Beta-ALPHA	0.666666667
EIF1AX	Beta-OMICRON	0.666666667
EIF4B	Beta-ALPHA	0.666666667
EIF6	Beta-OMICRON	0.666666667
EIF3L	Beta-OMICRON	0.615384615
EIF4B	Beta-OMICRON	0.615384615
EIF2S3	Unvaccinated-Vaccinated	0.6
EIF1AX	Unvaccinated-Vaccinated	0.545454545
EIF1	Unvaccinated-Vaccinated	0.5
EIF2S2	Unvaccinated-Vaccinated	0.461538462
EIF4G2	Unvaccinated-Vaccinated	0.461538462
EIF5A2	Unvaccinated-Vaccinated	0.461538462
EEF1B2	Unvaccinated-Vaccinated	0.428571429
EEF1D	Unvaccinated-Vaccinated	0.428571429
EIF5	Unvaccinated-Vaccinated	0.428571429
EIF5A	Unvaccinated-Vaccinated	0.428571429
MRRF	Unvaccinated-Vaccinated	0.428571429
EEF2	Unvaccinated-Vaccinated	0.4
EIF3I	Unvaccinated-Vaccinated	0.4
EIF3L	Unvaccinated-Vaccinated	0.4
EEF1A1	Unvaccinated-Vaccinated	0.375
EIF3G	Unvaccinated-Vaccinated	0.375
EIF1AY	Unvaccinated-Vaccinated	0.352941176
EEF1D	Beta-ALPHA	0.6
EEF1E1	Beta-OMICRON	0.6
EIF2B5	Beta-ALPHAE484K	0.6
EIF2B4	Beta-ALPHAE484K	0.545454545
EIF2S2	Beta-ALPHAE484K	0.545454545
EIF2S2	Beta-OMICRON	0.545454545
EIF3D	Beta-ALPHAE484K	0.545454545
EIF3D	Beta-ALPHA	0.545454545
EIF3H	Beta-ALPHA	0.545454545
EIF3K	Beta-OMICRON	0.545454545
EIF4B	Beta-ALPHAE484K	0.545454545
EIF2S2	Beta-ALPHA	0.5
EIF2B1	Unvaccinated-Vaccinated	0.333333333
EIF2B4	Unvaccinated-Vaccinated	0.333333333
EIF2S1	Unvaccinated-Vaccinated	0.333333333
EIF5B	Unvaccinated-Vaccinated	0.333333333
EIF2B3	Unvaccinated-Vaccinated	0.307692308
EIF2B5	Unvaccinated-Vaccinated	0.307692308
EIF3A	Unvaccinated-Vaccinated	0.307692308
EIF3J	Unvaccinated-Vaccinated	0.307692308
EIF4E	Unvaccinated-Vaccinated	0.307692308
MTOR	Unvaccinated-Vaccinated	0.307692308
EEF1G	Unvaccinated-Vaccinated	0.285714286
EIF3M	Unvaccinated-Vaccinated	0.285714286
EEF1DP3	Unvaccinated-Vaccinated	0.266666667
EIF3B	Unvaccinated-Vaccinated	0.266666667
EIF3C	Unvaccinated-Vaccinated	0.266666667
EIF3H	Unvaccinated-Vaccinated	0.266666667

EIF4B	Unvaccinated-Vaccinated	0.266666667
EEF1A2	Unvaccinated-Vaccinated	0.25
EIF2B2	Unvaccinated-Vaccinated	0.25
EIF3K	Unvaccinated-Vaccinated	0.25
EIF2A	Beta-ALPHA	0.444444444
EEF1D	Beta-ALPHAE484K	0.4
EEF1G	Beta-ALPHAE484K	0.4
EEF1G	Beta-ALPHA	0.4
EEF2	Beta-ALPHA	0.4
EIF2A	Beta-ALPHAE484K	0.4
EIF3A	Beta-ALPHA	0.4
EIF3C	Beta-ALPHA	0.4
EIF3H	Beta-ALPHAE484K	0.4
EIF4G1	Beta-ALPHA	0.4
EIF4G2	Beta-ALPHAE484K	0.4
EIF5A2	Beta-ALPHA	0.4
MRRF	Beta-ALPHA	0.4
EEF1A1	Beta-OMICRON	0.363636364
EEF2	Beta-ALPHAE484K	0.363636364
EIF2A	Beta-OMICRON	0.363636364
EIF3D	Beta-OMICRON	0.363636364
EIF3G	Beta-ALPHA	0.363636364
EIF3H	Beta-OMICRON	0.363636364
EIF4H	Beta-ALPHAE484K	0.363636364
EIF5A2	Beta-ALPHAE484K	0.363636364
EEF1D	Beta-OMICRON	0.333333333
EIF2A	Unvaccinated-Vaccinated	0.166666667
EIF3D	Unvaccinated-Vaccinated	0.142857143
EIF4G1	Unvaccinated-Vaccinated	0.142857143
EIF4H	Unvaccinated-Vaccinated	0.133333333
EIF2B4	Beta-ALPHA	0.222222222
EIF3B	Beta-ALPHA	0.222222222
EIF6	Beta-ALPHAE484K	0.222222222
EEF1A2	Beta-ALPHAE484K	0.2
EEF1A2	Beta-ALPHA	0.2
EEF1B2	Beta-OMICRON	0.2
EIF2S1	Beta-ALPHAE484K	0.2
EIF3A	Beta-ALPHAE484K	0.2
EIF3B	Beta-ALPHAE484K	0.2
EIF3C	Beta-OMICRON	0.2
EIF3E	Beta-OMICRON	0.2
EIF3G	Beta-ALPHAE484K	0.2
EIF4E	Beta-ALPHAE484K	0.2
EEF1G	Beta-OMICRON	0.181818182
EEF2	Beta-OMICRON	0.181818182
EIF3B	Beta-OMICRON	0.181818182
EIF3G	Beta-OMICRON	0.181818182
EIF5A2	Beta-OMICRON	0.181818182
